# Elevated IL-6R on CD4^+^ T cells promotes IL-6 driven Th17 cell responses in patients with T1R leprosy reactions

**DOI:** 10.1038/s41598-020-72148-7

**Published:** 2020-09-15

**Authors:** Chaman Saini, Rupesh K. Srivastava, Mohd. Tarique, Santosh Kurra, Neena Khanna, V. Ramesh, Alpana Sharma

**Affiliations:** 1 Department of Biotechnology, All India Institute of Medical Sciences, New Delhi, India; 2 Department of Biochemistry, All India Institute of Medical Sciences, New Delhi, India; 3 Department of Dermatology, All India Institute of Medical Sciences, New Delhi, India; 4 Department of Dermatology, Safdarjung Hospital, New Delhi, India

**Keywords:** Immunology, Microbiology, Diseases

## Abstract

Th17 cells play vital role during pathogenesis of leprosy reactions. Previously, we have reported that IL-23 is involved in Th17 cells differentiation. Subsequently, our group also showed that IL-6 induces Th17 cell differentiation along with TGF-β in leprosy reactions. Here, we next asked the question that whether IL-6 or IL-23 induced Th17 cells are different in nature? In this study, *Type 1 Reactions* (T1R) showed significantly (p < 0.001) higher percentage of IL-17A producing CD4^+^IL6R^+^ T cells as compared to non-reaction (NR) patients. Furthermore, recombinant IL-6, IL-23 and TGF-β promoted IL-17A secretion by CD4^+^IL6R^+^ T cells. Subsequently, IL-6R and IL-23R blocking experiments showed significantly (p < 0.002) down regulated IL-17A in T1R reaction as compared to NR leprosy patients. The present study for the first time establishes that pathogenic Th17 cells produce IL-17 in an IL-6 dependent manner in leprosy T1R reactions. Thus, present approaches that specifically target Th17 cells and/or the cytokines that promote their development, such as IL-6, TGF-β and IL-23A may provide more focused treatment strategies for the management of *Mycobacterium leprae* and its reactions.

## Introduction

Type 1 reaction (T1R) of leprosy can develop acute episodes of inflammation leading to severe morbidity, nerve damage and irreversible disability. T1R, classified as type IV hypersensitivity reaction mainly occurs during the course of Multi Drug Therapy (MDT) or after completion of MDT. In Indian scenario, approximately 25–30% borderline leprosy (BT, BB and BL) patients undergo in T1R, which is characterized by acute inflammation of pre-existing skin lesions or by the appearance of new lesions and/or neuritis^1^. These reactions are the consequence of dynamic nature of immune response against *Mycobacterium leprae* (*M. leprae*)*.* From the point of clearing bacteria, such increased cellular immune responses may be beneficial, because they promote bacterial killing mechanisms. However, the accompanying inflammation in and around the infected nerve tissue can result in severe and irreversible damage within few days. It is not clear how immune response initiates this spontaneous and natural T cell activation. During reactions, leprosy patients show increased lympho-proliferation against *M. leprae* antigens as well as increased expression and release of pro-inflammatory cytokines such as IL-1β, IL-2, IL-12, IFN-γ and TNF-α^2,3^. Some cytokines such as IP-10, TNF-α, IL-6, IL-10, IL-12, IL-17F and soluble receptors of IL-2 have also been reported in circulation^4–7^.

The role of Th1 and Th2 cells has been well studied in leprosy reactions. Recently a new avatar of pro-inflammatory cells called as Th17 which secrete IL-17 has also been reported in leprosy^8^. Previously, we showed that patients with non-polarized Th0 (IFN-γ^+^IL-4^+^) subset had increased percentage of IL-17^+^ cells which may constitute the third subset i.e. Th17 cells in leprosy patients who failed to show either Th1 and Th2 polarization. We previously reported that Th17 cells produces IL-17A, IL-17F and IL-21, thereby playing important role in the immunopathology of tissue^8^. Subsequently, we have also reported that double IL17A^+^/F^+^ cells recruit to IL-17 producing neutrophils in reversal reactions of leprosy^9^. On the other hand TGF-β producing FOXP3^+^ Treg cells have role in maintaining tolerance and inflammation regulation in leprosy^10^. Th17 and Treg cells regulate immune system and are also activated by various cytokines, such as IL-1β, IL-6, IL-23 and TGF-β. The differentiation of naive T cells into Th17 cells is regulated by several mechanisms. Mainly, it has been reported that TGF-β and IL-6 coordinately induce Th17 differentiation^11,12^ via induction of transcription factor ROR-γt, which is a downstream target of signal transducer and activator of transcription 3 (STAT3)^13–15^. Saini et al.^8^, reported that IL-23 is also identified as an essential cytokine for Th17 cells differentiation in leprosy diseases. Furthermore, our group has also reported that rIL-23 modulates the plasticity of Tregs in leprosy patients which are converted into Th17 like cells^16^. Moreover, aryl hydrocarbon receptor (AHR) contributes to the Th17 cells differentiation through a cytokine-independent mechanism^17^. Among these differentiating cytokines, TGF-β is essential for polarizing naive T cells towards Th17 and Treg cells. Interestingly, our previous report showed synergistic effect of TGF-β with the pro-inflammatory cytokine IL-6 in inducing Th17 differentiation^18^. Moreover, IL-23 is also involved in Th17 differentiation in non-reaction (NR) leprosy patients. Surprisingly, the association of IL-23R and not IL-6R with IL-17^+^ cells in NR leprosy patients is not well studied. IL-6R and IL-23R are key players in the development and maintenance of Th17 cells^19^. A recent study demonstrated increased percentage of CD4^+^ T cells expressing IL-6R in chronic hepatitis B patients and higher levels of IL-17 upon stimulation with the HBV core antigen (HBcAg) in vitro^20^. IL-6 cytokine has the ability to polarize Th1/Th2 cells balance towards Th2 cell subset. Several studies have shown that murine naïve CD4^+^ T cells differentiate into Th17 cells via simultaneous treatment with IL-6 and low doses of TGF-β^21^.

Our second study on leprosy reactions demonstrated that elevated expression of IL-6 and lower TGF-β in leprosy reaction initiated synergistic effect in differentiation of Th17 cells^18^. In brief emergencies in *reversal reactions* is increased by Th17 cells which may be triggered by subsequently decreased Treg cell activity through low TGF-β producing FOXP3^+^ Treg cells. The synergetic effect of down regulated TGF-β and upregulated IL-6 in both reactions may play an important role in the balance of Treg and Th17 cell differentiation and thereby lead to the immunopathology associated with leprosy reactions^18^. Th17 and Treg cells are key players in immunopathology of leprosy reaction and leprosy as reported by us and others^8,10,18,22,23^. Their role in leprosy reactions and the differentiation and signaling pathways of these cells are yet to be fully determined. Therefore, by elucidating the molecular mechanisms that link Th17 differentiation and immuno-pathogenesis in leprosy reactions, a novel therapeutic strategy could be provided. Various kinds of cytokines and receptor can positively or negatively regulate the development of leprosy reactions. In contrast, interferon (IFN)-γ, a Th1 cytokine, is considered a positive regulator of leprosy, as disruption or low production of IFN-γ enhances the severity in leprosy^4^. These findings indicate that cytokine network determines the progression or regression of leprosy^24^. In the present study, using IL-6 receptor (IL-6R)-blocking antibody, we examined the effects of IL-6 blockade on leprosy reactions, in light of Th17 cell differentiation. Consequently, we report that IL-6 is essential for the initiation of leprosy reactions, rather than its progression, through Th17 differentiation. The present study thus for the first time establishes that specifically targeting Th17 cells along with its cytokine IL17 may provide more focused treatment strategies for the management of *M. leprae* and its reaction.

## Materials and methodology

### Patient recruitment and ethics statement

A total of 56 new patients (Untreated; without MDT and steroid) were investigated. Samples were obtained from patients who were clinically and histologically proven for leprosy type according to Ridley–Jopling classification^25,26^. All methods were carried out in accordance with relevant guidelines and regulations of the ethical permission (Ref. No. IEC-275/02.06.2017, RP-25/2017), AIIMS, New Delhi. Informed written consent was taken prior to recruitment (Table 1).

**Table 1:** Demographic details of leprosy patients included from Department of Dermatology, AIIMS, New Delhi.

S. no	Leprosy classification	No of cases investigated	Sex		Age range
			M	F	
1	Borderline tuberculoid (BT; NR)	29	18	11	18–30
2	Reversal reaction (RR) or Type I reaction (T1R)	29	19	10	19–36
	Total	58	27	21	

Patients were typed on the basis of Ridley–Jopling classification^25,26^, M; male, F; female.

### Isolation of peripheral blood mononuclear cells (PBMCs) from blood samples

Six ml heparinized venous blood samples were layered on Ficoll-Hypaque (Sigma Aldrich, USA) after diluting with 1:1 in RPMI 1640. PBMCs were isolated by density gradient centrifugation at 800×g for 20 min. Cells in the interphase were collected into a new tube and washed thrice in sterile 1 × HBSS by centrifugation at 1,600 rpm for 10 min. After the last wash cells were re-suspended in RPMI 1640 along with 10% FBS (Sigma, USA), cell viability and enumeration were estimated by 0.2% trypan blue exclusion using hemocytometer.

### Cell culture

2 × 10^6^ cells/ml were stimulated with or without *Mycobacterium leprae sonicated antigen* (MLSA; 10 μg/ml) and recombinant-IL-6 (10 ng/ml; Peprotech NJ, USA), r-TGF-β (2.0 ng/ml, Peprotech NJ, USA) and r-IL-23 (6.0 ng/ml, R&D, USA) were added with different combinations. Cultures were also stimulated with phytohemagglutinin (PHA; 5 μg/ml, Sigma, USA). Cells were stimulated in tissue culture media containing 10% FBS (Sigma, USA) and RPMI 1640 (Gibco, USA) and incubated in 5% CO_2_ incubator at 37 °C for 48 h. In our previous study we already performed and standardized stimulation at various time points of 6, 24 and 48 h stimulation with MLSA in leprosy patients and we observed that Th17 related genes, phenotypic characterization, cytokine release etc. in ex-vivo antigen stimulated PBMC cultures were optimum at 48 h^8^. After these cells were used for FACS analysis and other experiments.

### IL-6R and IL23R blockade treatment

PBMCs from non-reactions (NR) and reactions (T1R) leprosy patients were incubated in RPMI-1640 medium alone or stimulated with MLSA (10 μg//ml) for 48 h, neutralizing antibody against IL-6R and IL-23R (R&D systems, Minneapolis, MN) were added at a concentration of 0.5 and 2.0 µg/ml respectively, 30 min before the addition of MLSA. Prior to 6 h cells harvesting, GolgiStopTM protein transport inhibitor (BD, biosciences, USA) was added for intracellular cytokine staining and analyzed by flow cytometry. Moreover, a separate culture setup without GolgiStopTM was also performed for IL-17 protein estimation through ELISA.

### Cell surface and intracellular staining for flowcytometry analysis

All anti-human antibodies were obtained by BioLegend, USA. Staining was done within 1 h after harvesting and washing three times as above and cell viability determined. In brief, for cell surface staining, 0.1 × 10^6^ cells/50 µl in staining buffer were incubated with a cocktail containing anti-human antibodies against CD3 (APCH7, clone SK7), CD4 (PerCP/Cy5.5, clone HIT8a and FITC, clone RPA-T4), IL-6R (PE-Cy7, cloneUV4) and IL-23R (PE, clone 218213) for 45 min at 4 °C. After cell surface staining, cells were incubated with BD Cytofix/CytopermTM buffer for 20 min at room temperature. The cells were washed twice resuspended in stain buffer and incubated with anti-human fluorochrome labeled antibodies against IL-17A (APC, clone BL168). Subsequently, after the surface staining 1XFOXP3 buffer A for 10 min at room temperature; cells were then washed twice and permeabilized with buffer C for 30 min at room temperature. The cells were further washed twice, resuspended in stain buffer and incubated with anti-human fluorochrome labeled antibodies against IL-17A (APC), FOXP3 (PE, clone 259D), TGF-β (PercpCy5.5, clone TW4-2F8) and IFN-γ (PE-Cy7, clone 4S.B3) at room temperature for 30 min in the dark, followed by two washes as before and resuspended in 500 µl with appropriate FMO and isotype controls. Multi-color flow cytometry analysis was performed on FACS canto with DIVA software (BD Bioscience, USA).

### CD4^+^ T cells sorting by FACS

The following anti-human flurochrome labeled antibodies against CD3 (Per cpcy-5.5, clone: UCHT1), CD4 (APC-H7, clone: SK3), CD8 (PE-Cy7, clone: RPA-T8), CD25 (FITC, clone: M-A251) and FOXP3 (APC, clone: 259D/C7) for cell sorting by flow cytometry on BD FACS AriaTM as per manufacturer’s instructions. Using 48 h MLSA stimulated PBMCs for staining by above anti-human antibodies (see “Methodology”) with relevant isotype controls, accordingly we set up the following gating strategy for sorting (Supplementary Fig. 1): (a) Lymphocytes were gated as FSC vs SSC (b) FSC vs CD3 and (c) CD4^+^ vs CD8^+^ T cells. After gating, CD4^+^ T cells were further gated as CD25^+^FOXP3^+^ and CD25^neg^FOXP3^neg^ cells, deriving each gate as a daughter of the gate set up in the previous step as FSC vs. SSC. Two 15 ml centrifuge tubes were prepared by adding 2 ml complete medium as collection tubes for sorting and recorded ~ 20,000 events from each staining control sample. Gates, if necessary were adjusted, based upon the staining controls. Once the gates were standardized and satisfactory, samples were loaded, and sorting performed. Sorted cells were resuspended in complete medium. To assess the purity of the sorted cells, a small aliquot was run on flowcytometry, under the same template as was used for the sorting. Purity of more than 96.2% was routinely observed. The sorted CD4^+^ T cells were later cryo-preserved for qPCR analysis.

### RNA isolation and reverse transcriptase PCR reaction

RNeasy Mini Kit (Qiagen, USA) used for RNA isolation from sorted CD4^+^CD25^neg^FOXP3^neg^ cells according to manufacturer’s instructions. Nanodrop spectrophotometer (Nanodrop Technologies, USA) was used for quantification of RNA. RNA purity at 260/280 from 1.8 to 2.0 was considered to be best. RNA (28S and 18S) was also checked for quality, RIN value of ≥ 7 was considered to be optimum by using Bioanalyzer (Agilent Technologies, Singapore). For reverse transcriptase PCR reaction, 500 ng total RNA was transcribed into cDNA using RT First strand kit (SA Biosciences, USA). RT-PCR was performed according to the manufacturer’s instructions and cDNA stored at − 20 °C till further use.

### Real time PCR (q-PCR) array

Commercially customized PCR array (Qiagen, USA) was employed as per manufacturer’s instructions with the following primers of *IL-6R*, *IL23R*, *IL-1β*, *Stat3*, *TNF-α*, *CCL22*, *RORC*, *JAK1*, *IL-2*, *IL-17F*, *JAK2*, *CCL20*, *IL-17D*, *IL-23*, *IL-17A*, *IL-23R*, *IL-10*, *IL-17C*, *IL-6*, *IL-25*, *IL-21*, *IL-22* for gene expression. 500 ng of cDNA was used per array containing the ready to use PCR master mix. These were then subjected to q-PCR (ABI 7000, Applied Bio-systems, Singapore) for 2 h. Threshold cycle (Ct) values of specific gene were normalized with housekeeping genes (*β2M*, *HPRT1*, *RPL13A*, *GAPDH* and *ACTB*) and expressed as ΔCt (Table 2). SA Biosciences (Qiagen) online software (http://pcrdataanalysis.sabiosciences.com) was used for creating heat maps showing intensity of expression of genes. Patients with T1R were compared with NR patients.

**Table 2:** In situ gene expression (Mean ± SD) of Th17 signature and associated molecules in CD3^+^CD4^+^ T cells isolated from 48 h MLSA stimulated PBMCs.

S. no	Gene	Accession number	Gene expression (ΔCt) Mean ± SD		P value
			T1R (19)	NR(19)	
1	*IL-1β*	*Hs.126256*	2.25 ± 1.3	2.97 ± 1.3	ns
2	*Stat3*	*Hs.463059*	2.55 ± 0.8	2.97 ± 0.6	p < 0.04
3	*TNF-α*	*Hs.241570*	3.91 ± 1.3	4.62 ± 0.8	ns
4	*CCL22*	*Hs.534347*	3.13 ± 1.0	4.82 ± 1.2	p < 0.0001
5	*RORC*	*Hs.256022*	3.98 ± 2.1	7.07 ± 2.2	p < 0.0001
6	*JAK1*	*Hs.207538*	2.04 ± 0.6	2.62 ± 0.3	p < 0.005
7	*IL-2*	*Hs.89679*	4.29 ± 1.7	8.12 ± 3.6	p < 0.0003
8	*IL-17F*	*Hs.272295*	4.1 ± 1.8	8.0 ± 3.3	p < 0.0001
9	*JAK2*	*Hs.656213*	2.77 ± 1.1	3.92 ± 0.9	p < 0.001
10	*CCL20*	*Hs.75498*	3.54 ± 1.6	6.42 ± 2.1	p < 0.0001
11	*IL-17D*	*Hs.655142*	6.8 ± 2.5	10.8 ± 2.7	p < 0.0001
12	*IL-23A*	*Hs.98309*	3.38 ± 1.7	6.22 ± 1.7	p < 0.0001
13	*IL-17A*	*Hs.41724*	4.68 ± 2.6	9.02 ± 4.1	p < 0.0002
14	*IL-23R*	*Hs.677426*	4.01 ± 2.9	7.87 ± 3.7	p < 0.0005
15	*IL-10*	*Hs.193717*	3.32 ± 1.4	4.77 ± 1.0	p < 0.0005
16	*IL-17C*	*Hs.278911*	4.87 ± 2.1	7.77 ± 2.7	p < 0.0006
17	*IL-6R*	*Hs.709210*	2.95 ± 1.0	3.72 ± 0.6	p < 0.003
18	*IL-6*	*Hs.654458*	3.79 ± 1.5	6.82 ± 2.3	p < 0.0001
19	*IL-25*	*Hs.302036*	5.26 ± 2.6	9.52 ± 3.5	p < 0.0002
20	*IL-21*	*Hs.567559*	3.64 ± 2.5	7.32 ± 3.1	p < 0.0003
21	*IL-22*	*Hs.287369*	3.45 ± 2.7	8.02 ± 4.0	p < 0.0001

Classification of patients according to Ridley–Jopling^25,26^ as in legend to Table 1.*p < 0.01, **p < 0.001, ***p < 0.0001 by two tailed Mann–Whitney test. P < 0.05 was considered significant, ns = non significant.

### Estimation of cytokines by ELISA

For IL-17A estimation, sandwich ELISA (Ready Set Go, eBioscience, USA) was performed as per manufacturer’s instructions. Briefly, at 4 °C after overnight coating (un-conjugated) with 100 µl/well capture antibody in 96-well plates (Nunc, USA), was performed followed by 3× wash with washing buffer, and subsequently blocked with 1× assay diluent at room temperature for 1 h. After blocking, subsequently five times washing was performed with 1× washing buffer. 100 µl/well of 48 h stimulated PBMC culture supernatants were added in triplicate and incubated for another 2 h at room temperature. After incubation, washing was done and 100 µl/well of detection antibody (biotin-conjugated) diluted in 1× Assay Diluent (anti-mouse antibody) was added, and plates were further incubated at room temperature for 1 h. After 5 times washing, 100 µl/well of Avidin-HRP diluted in 1× assay diluent was added and incubated at room temperature for 30 min. Color development step was done with peroxidase color substrate TMB (Tetra Methyl Banzedine). After developing, the plate reaction was stopped by stopping solution containing 1 N H_2_SO_4_. The optical density (OD) was taken at 450 nm.

## Results

Isolated CD4^+^ T helper cells were investigated in 19 of each Type 1 reactions (T1R) patients and non-reactions (NR) leprosy patients by Real Time PCR (*qPCR*) for RNA estimation. 48 h, MLSA stimulated PBMCs cultures, were studied in patients with *Type 1 reactions* (T1R, n = 10) and non-reactions (BT, n = 10) leprosy patients and further evaluated by flow cytometry for Th17 cell characterization. Furthermore, ELISA was performed for measuring cytokines in culture supernatants. The enrolled patients details of the T1R reaction and NR clinical groups of leprosy are listed in Table 1.

### Hierarchical clustering identified inflammation associated genes correlation with *IL-6R*

**Figure 1:**
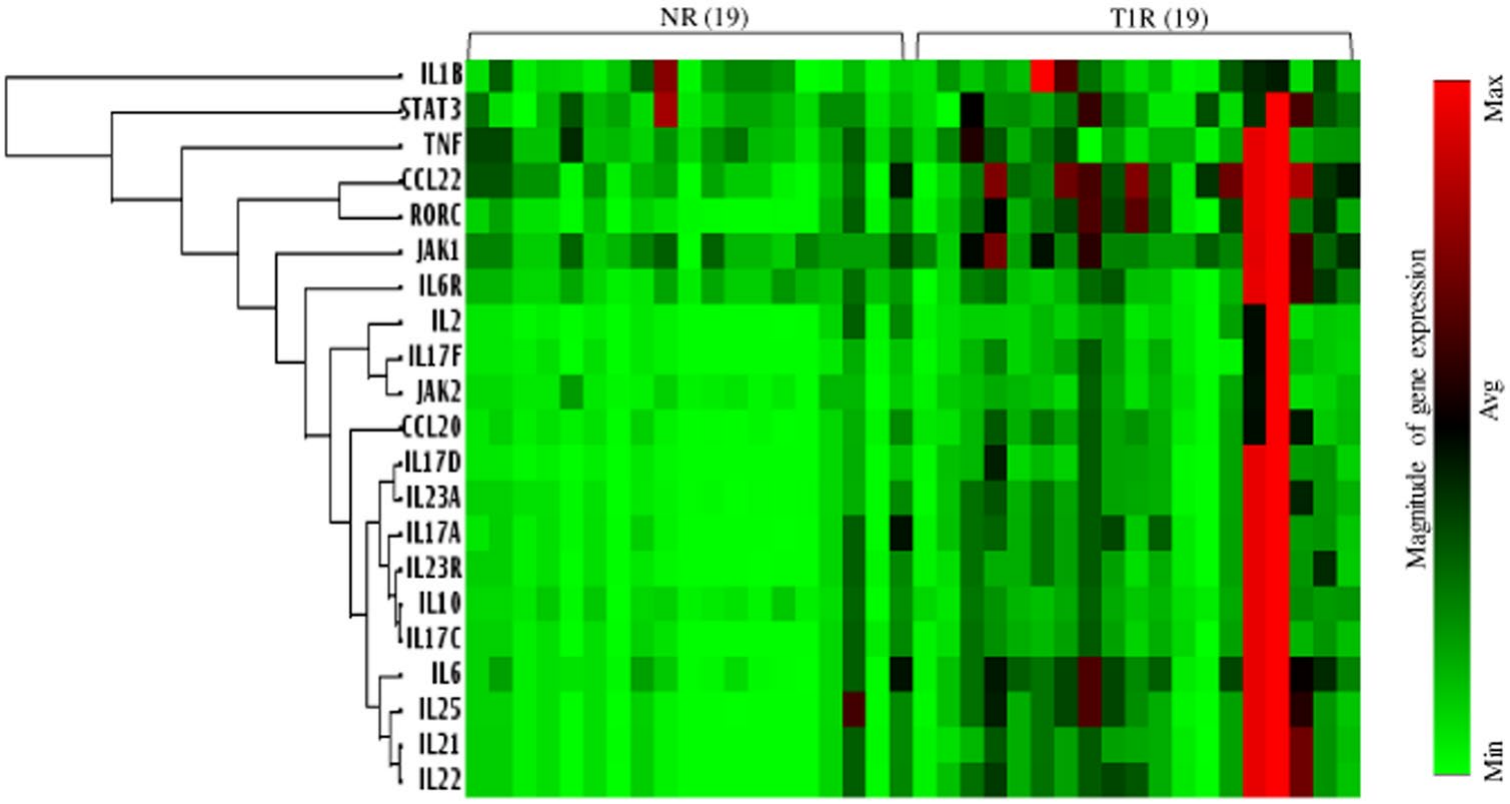
Gene expression of Th17 cells signatures and associated molecules in isolated CD4^+^ T cells. Figure showing Hierarchical clustering of heat map showing a correlation of IL-6R and IL-6 associated inflammatory markers (see also “Methodology” and Table 2).Groups of genes were associated with hierarchical clustering using the complete-linkage clustering (pcrdataanalysis.sabiosciences.com). Each horizontal row represents the same gene product and each vertical row the same patient. The fluorescence range from high (red) to low (green) is indicated by the colored bar and fluorescence intensity/gene expression. In general, T1R patients show higher expression of several genes representing, cytokines, cytokine receptors, and chemokines as compared to NR patients (see also Table 2). Abbreviations: Mann Whitney T-test was performed for the statistical difference, p < 0.05 value was considered as significant. (); Parenthesis shows patients number, NR; Non-reactions, T1R; Type 1 reactions). (For interpretation of the references to color in this figure legend, the reader is referred to the web version of this article).

**Figure 2:**
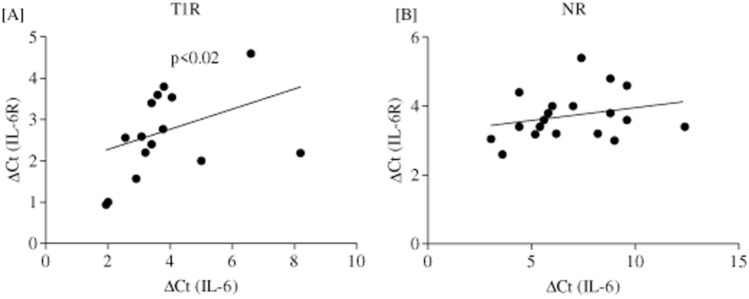
IL-6R and IL-6 gene expression showed positive correlation in T1R patients. Figure shows significant correlation of *IL-6R* and *IL-6* gene expression in (**A**) T1R (19) and (**B**) NR (19) patients. Figures in parenthesis indicate number of subjects studied. *P* < 0.05 was considered as a significant value by two-tailed Spearman test for correlation. Abbreviations: Δ*C*t, threshold cycle (delta Ct) of real time PCR; Mann Whitney T-test was performed for the statistical difference, p < 0.05 value was considered as significant. (); Parenthesis shows patients number, NR; Non-reactions, T1R; Type 1 reactions.

Gene expression of isolated CD4^+^ T cells of leprosy patients (NR vs T1R) were analyzed for 21 signature genes of Th17 lineage and associated molecules with a rationale to understand the differences between the immunopathology/inflammation associated with localized T1R reactions via qPCR assay (Fig. 1). Heat maps of gene expression showed significantly high intensity in T1R as compared to NR patients. Table 2 provides data on the statistical significance of the fold changes in gene expression in patients with these two reaction types. Further, we analyzed gene expression of Th17 associated markers such as *IL-1β*, *Stat3*, *TNF-α*, *CCL22*, *RORC*, *JAK1*, *IL-2*, *IL-17F*, *JAK2*, *CCL20*, *IL-17D*, *IL-23*, *IL-17A*, *IL-23R*, *IL-10*, *IL-17C*, *IL-6R*, *IL-6*, *IL-25*, *IL-21,* and *IL-22* by hierarchical clustering to show similar and distinct genes into independent groups (for complete list see “Methodology”). Of interest, all markers were significantly (range: p < 0.001–0.0004) upregulated in T1R as compared to NR leprosy patients. Although, among the T1R patients, two of them showed high intensity gene expression as they showed clinically erythematous plaques with hyperesthesia and tenderness. Surprisingly, hierarchical clustering identified 3 groups with IL-6R gene expression with Th17 cells markers. These genes have already been established for Th17 development (*IL-6*, *IL-25*, *IL-21 and IL-22*), inflammation (*IL-17C*, *IL-10*, *IL-23R*, *IL-17A*, *IL-23A*, *IL-17D*) along with migratory and signaling (*CCL20*, *JAK2*, *IL-17F and IL-2*) molecules. Taken together these data point that *IL-6* gene expression showed significant (p < 0.006) positive correlation with *IL-6R* in T1R patients. On the other hand, *IL-6* and *IL-6R* we found to have no correlation in NR patients (Fig. 2). Table 3 lists correlation between *IL-6R* and *IL-23R* gene expression with other Th17 related inflammatory markers. Moreover, *IL-6R* gene expression was also found to have significant (p < 0.01-p < 0.005) positive correlation with *IL-17* isomers in T1R but not in NR patients. On the other hand, IL-23R showed significant (p < 0.0001) positive correlation with IL-17 isomers in NR but not in T1R patients. Surprisingly, *IL-17C* showed positive correlation in both T1R and NR patients (Table 3).This may be due to variation in kinetics of secretion and thus further study is warranted.

**Table 3:** Correlation between IL-6R and IL-23R gene expression and other Th17 related inflammatory markers.

S. no	Gene	IL-6R				IL-23R			
		NR		T1R		NR		T1R	
		Spearman-r	p < value	Spearman-r	p < value	Spearman-r	p < value	Spearman-r	p < value
1	*IL-1β*	0.23	ns	0.08	ns	0.05	ns	0.66	p < 0.003
2	*IL-10*	0.36	ns	0.44	ns	0.63	p < 0.003	0.87	p < 0.0001
3	*IL-23R*	0.20	ns	0.35	ns	–	–	–	–
4	*IL-23A*	0.34	ns	0.43	ns	0.90	p < 0.0001	0.87	p < 0.0001
5	*IL-6R*	–	–	–	–	0.24	ns	0.43	ns
6	*IL-6*	0.3	ns	0.6	p < 0.006	0.79	p < 0.0001	0.79	p < 0.0001
7	*JAK1*	0.47	p < 0.03	0.50	p < 0.03	0.29	ns	0.70	p < 0.001
8	*JAK2*	0.55	p < 0.01	0.17	ns	0.60	p < 0.006	0.61	p < 0.009
9	*Stat-3*	0.41	ns	0.61	p < 0.008	0.10	ns	0.66	p < 0.003
10	*IL-17A*	0.17	ns	0.61	p < 0.005	0.90	p < 0.0001	0.24	ns
11	*IL17F*	0.17	ns	0.59	p < 0.007	0.87	p < 0.0001	0.26	ns
12	*IL-17C*	0.28	ns	0.47	p < 0.04	0.91	p < 0.0001	0.57	p < 0.01
13	*IL-17D*	0.21	ns	0.54	p < 0.01	0.90	p < 0.0001	0.31	ns
14	*RORC*	0.23	ns	0.21	ns	0.92	p < 0.001	0.64	p < 0.004
15	*IL-21*	0.22	ns	0.39	ns	0.96	p < 0.0001	0.78	p < 0.0002
16	*IL-22*	0.19	ns	0.44	ns	0.92	p < 0.0001	0.71	p < 0.001
17	*CCL20*	0.13	ns	0.27	ns	0.9	p < 0.0001	0.62	p < 0.007
18	*CCL22*	0.33	ns	0.51	p < 0.03	0.78	p < 0.0001	0.43	ns
19	*IL-25*	0.25	ns	0.43	ns	0.91	p < 0.0001	0.79	p < 0.001
20	*IL-2*	0.21	ns	0.19	ns	0.91	p < 0.0001	0.81	p < 0.0001
21	*TNF-α*	0.53	p < 0.01	0.22	ns	0.43	ns	0.70	p < 0.002

Classification of patients according to Ridley–Jopling^25,26^ as in legend to Table 1. Significant correlation calculated by two tailed Spearman test. P < 0.05 was considered significant, ns = non significant.

### Type 1 reactions showed high CD4^+^IL6R^+^ T cells

**Figure 3:**
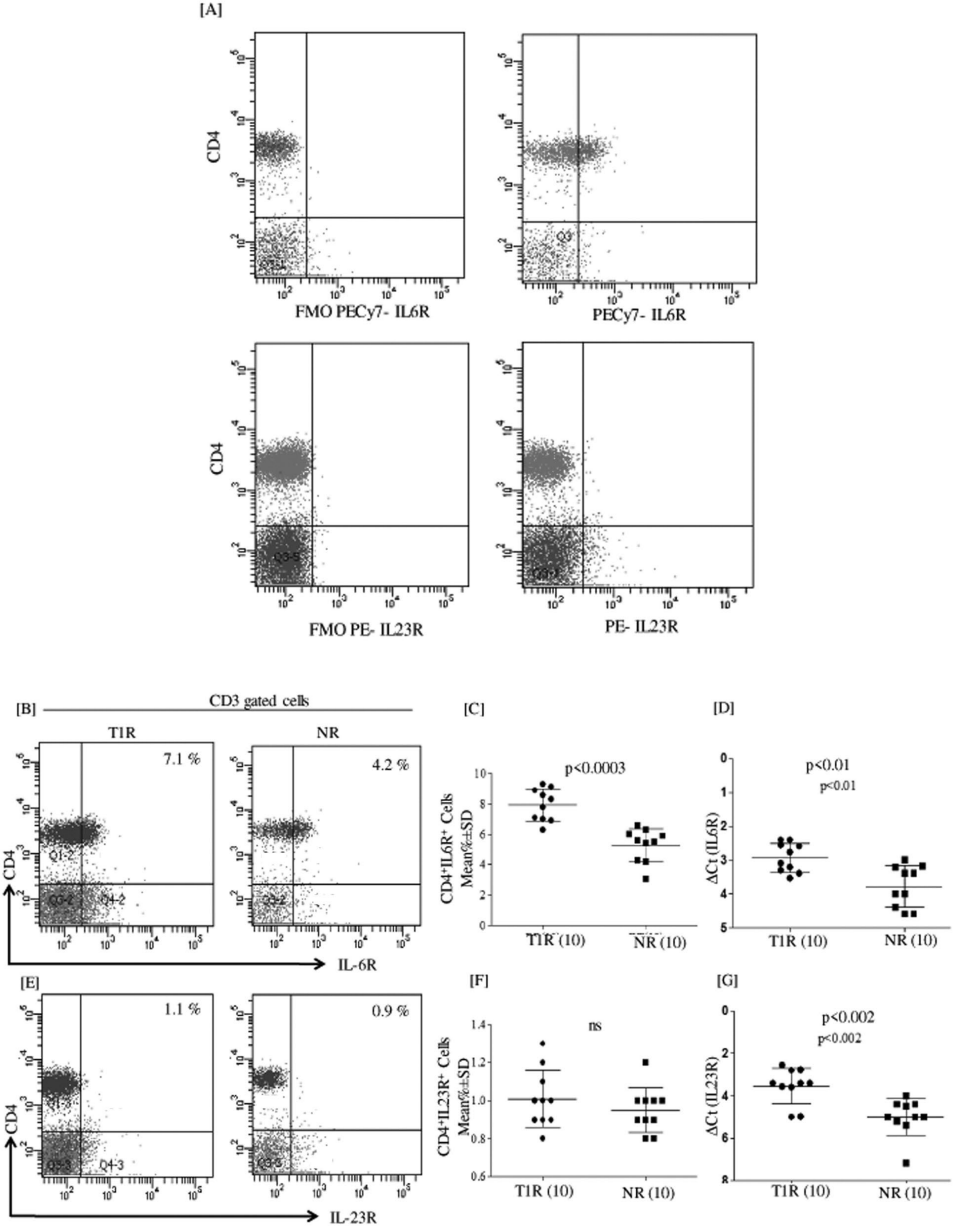
IL-6R showed high expression in T1R patents. (**A**) dot plot is showing FMO control for IL6R^+^ and IL23R^+^ with respective flurochrome. (**B**) Dot plot is showing CD3^+^ gated CD4^+^IL-6^+^ and (**E**) CD4^+^ IL-23R^+^ T cells in T1R and NR patients respectively in 48 h MLSA stimulated PBMC cultures. (**C**) Scattergram showing mean% ± SD of IL-6R^+^ cells were significantly (p < 0.0003) upregulated cells in T1R as compared to Non-reactions leprosy patients. (F) IL-23R^+^ cell not showing any significant change as compared to T1R and NR patients (**D**, **G**) Representative Scatergram showing gene expression of *IL-6R* and *IL-23R* in T1R and NR patients respectively. Abbreviations: Mann Whitney T-test was performed for the statistical difference, p < 0.05 value was considered as significant. (); Parenthesis shows patients number, NR; Non-reactions, T1R; Type 1 reactions), MLSA; *Mycobacterium leprae* sonicated antigen.

Figure 3A flowcytometry dot plot is showing FMO control for IL6R^+^ and IL23R^+^ with respective flurochrome. Figure 3B,E shows analyzed 48 h MLSA stimulated PBMC by flow cytometry in T1R and NR patients. Of interest, we have also compared in-vitro MLSA stimulated with unstimulated cultures to know how IL-6R and IL-23R expression respond under these conditions. Interestingly, we didn’t find any significant difference (data not shown). Dot plot shows CD4^+^IL-6R^+^ (Fig. 3B) and IL23R^+^ cells (Fig. 3E). CD4^+^IL-6R^+^ cells (Fig. 3C) were significantly high (p < 0.0003) in T1R (mean% ± SD; 7.9 ± 1.0) as compared to NR (5.2 ± 1.0) patients. However, CD4^+^IL23R^+^ (Fig. 3F) cells were detected without any difference between T1R and NR leprosy patients. Moreover, we further analyzed co-expression of IL-6R^+^ and IL-23R^+^ on CD4^+^ cells but no significant co-expression was detected (data not showed). Of interest, gene expression of *IL-6R* and *IL-23R* in T1R and NR patients in 48 h MLSA stimulated PBMC (Fig. 3D,G) showed a significant increase (p < 0.01, p < 0.002) in T1R as compared with NR leprosy patients. *IL-6R* and *IL-23R* showed high delta threshold cycles (ΔCt) (mean ± SD; 2.9 ± 0.4) and (3.5 ± 0.8) in T1R group as compared to (3.7 ± 0.6) and (5.0 ± 0.8) NR patients respectively.

### Higher production of IL-17A by CD4^+^IL6R^+^ T cells in T1R

**Figure 4:**
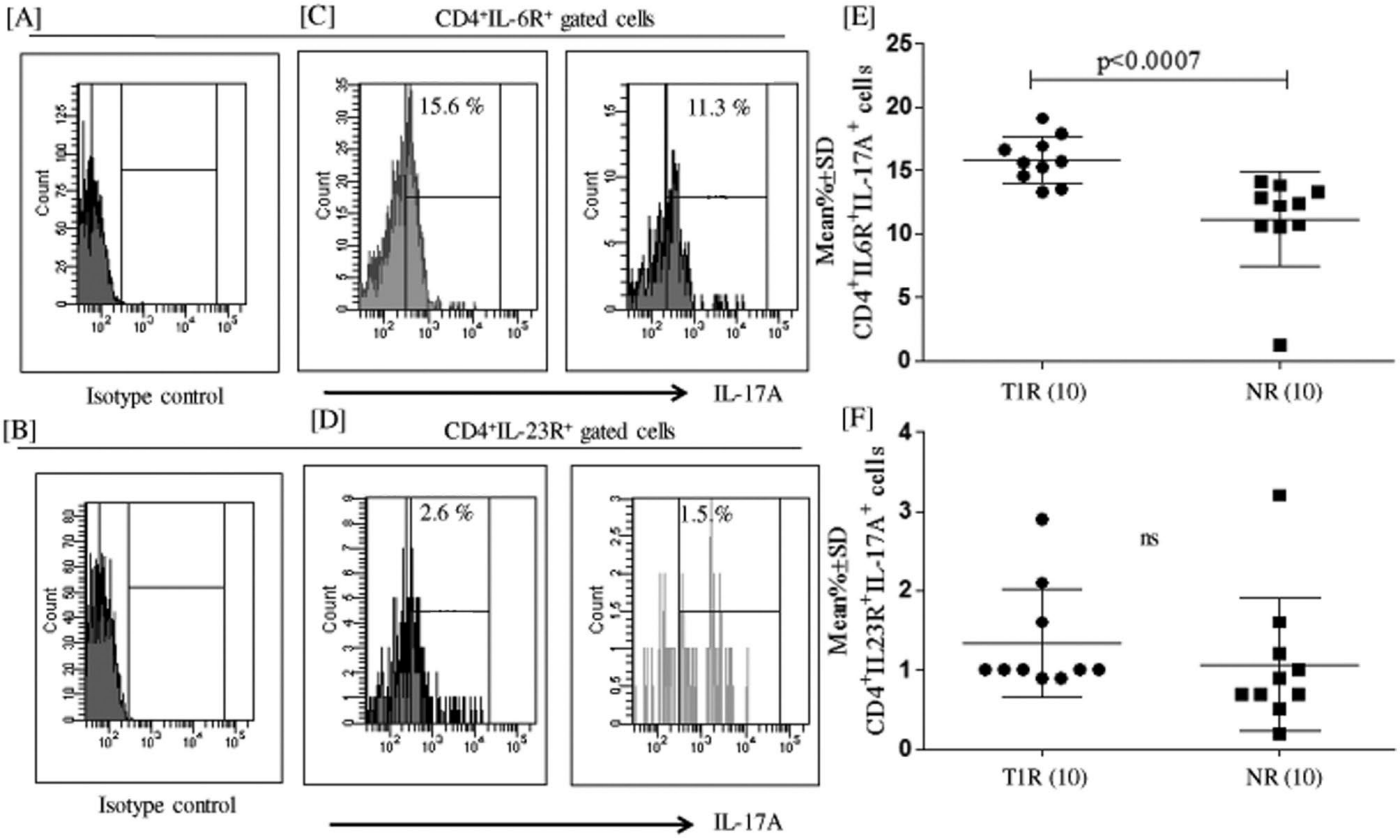
Higher expression of IL-6R^+^IL-17A^+^ cells in T1R. Histogram (**A**, **B**) is showing isotype control for IL-17 (APC) flurochrome. (**C**) Histogram of flowcytometry shows CD4^+^IL-6R^+^ IL17A^+^ and (**D**) CD4^+^IL-23R^+^ IL17A^+^ cells of 48 h M*. leprae* sonicated antigen (MLSA) stimulated PBMC by flow cytometry. (**E**) and (**F**) showed scatergram of CD4^+^IL-6R^+^ IL17A^+^ and CD4^+^IL-23R^+^ IL17A^+^ cells in T1R and BT patients respectively in 48 h MLSA stimulated PBMC. The CD4^+^IL-6R^+^IL-17A^+^ cells were significantly (p < 0.0007) upregulated in T1R as compared to Non-reactions leprosy patients. Abbreviations: Mann Whitney T-test was performed for the statistical difference p < 0.05 value was considered as significant. (); Parenthesis shows patients number, NR; Non-reactions, T1R; Type 1 reactions), MLSA; *Mycobacterium leprae* sonicated antigen.

We further analyzed CD4^+^IL-6R^+^IL-17A^+^ and CD4^+^IL-23R^+^IL-17A^+^ T cells in 48 h MLSA stimulated PBMCs in both T1R and NR patients. Figure 4 represents high percentage (Mean ± SD) of CD4^+^IL6R^+^IL-17A^+^ T cells with significantly (p < 0.0007) higher expression of IL-6R^+^ Th17 cells in T1R (15.8 ± 1.8) leprosy patients as compared to NR (11.1 ± 3.7) leprosy patients. On the other hand, IL-23R^+^IL-17A^+^ T cells were found to be insignificant between T1R (1.3 ± 0.6) and NR (1.0 ± 0.8) leprosy patients.

### IL-6 and IL-23 induce IL-17A production via TGF-β independent manner in T1R leprosy patients

**Figure 5:**
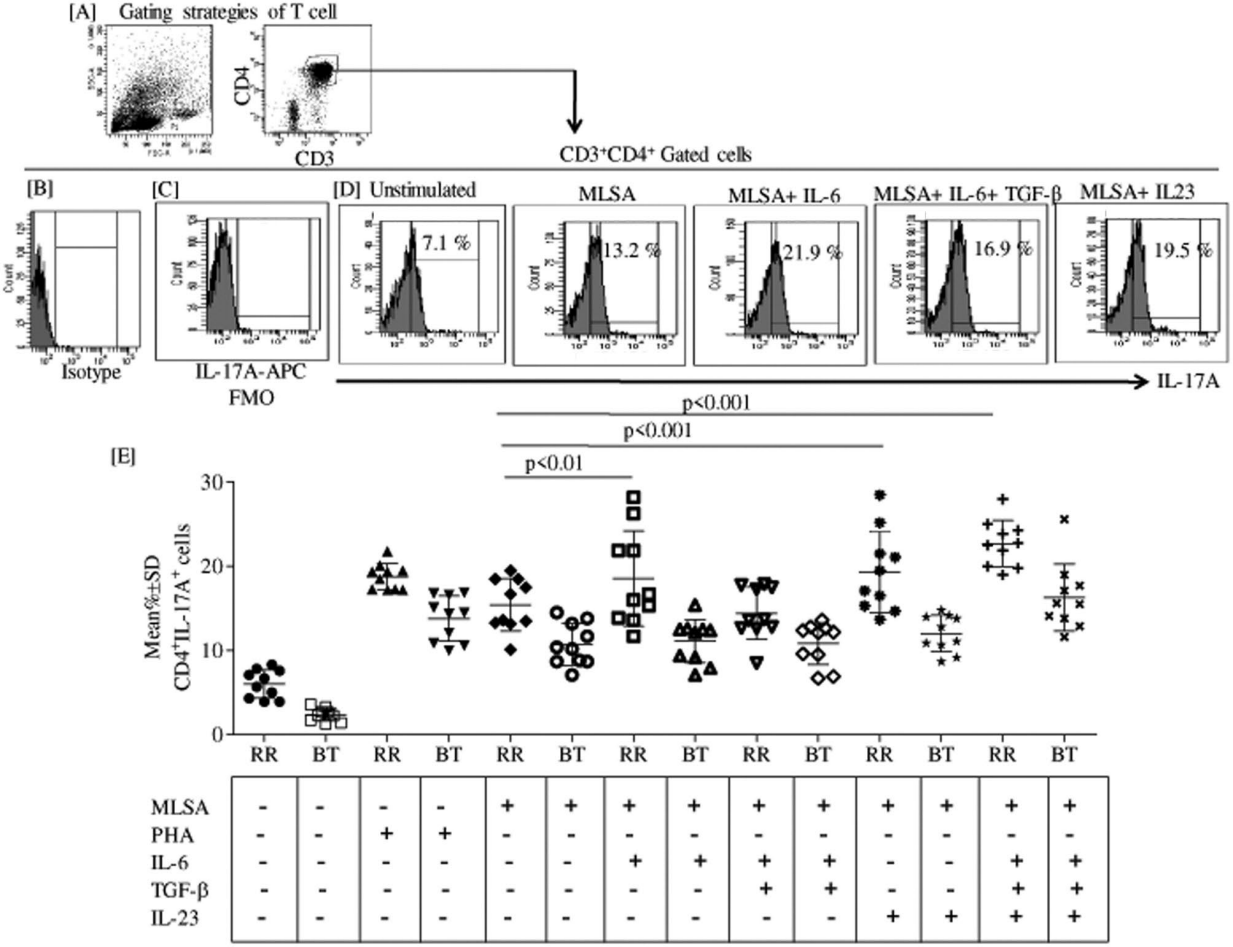
IL-6 and IL-23 induces IL-17 productionnot TGF-β in T1R leprosy patients. (**A**) shows lymphocytes gated as FSC vs SSC and CD3^+^ vs CD4^+^ T cells. After gating, CD3^+^CD4^+^ T cells were further gated as IL-17A^+^ cells. (**B**) Shows isotype control and (**C**) FMO control respectively for APC labeled IL-17A. Representative (**D**) shows histogram of flow cytometry showing 48 h stimulated MLSA and diffrent combinations of recombinants IL-6, IL-23 and TGF-β stimulated CD4^+^IL17A^+^ cells. (**E**) Scatter plot is showing mean% ± SD of IL-17A producing CD4^+^ cells by diffrent combinations in T1R (10) and NR (10) patients. Abbreviations: Mann Whitney T-test was performed for the statistical difference p < 0.05 value was considered as significant. NR; Non-reactions, T1R; Type 1 reactions), MLSA; *Mycobacterium leprae* sonicated antigen.

After checking the status of IL-6R^+^ and IL-23R^+^ Th17 cells in reactional states, we were next interested in the secretion status of IL-17 via Th17 cells with or without MLSA, r-IL-6, r-TGF-β and r-IL-23 in both T1R and NR leprosy patients. Figure 5A shows lymphocytes gated T cells and Fig. 5B showing isotype control and Fig. 5C FMO control respectively for APC labeled IL-17A. Representative, flowcytometry histograms showed comparison of IL-17A in 48 h stimulated PBMC with different combination of recombinant proteins (Fig. 5D). Scattered plot showed significantly (p < 0.001) high percentage (Mean ± SD) of IL-17A in IL-6 (18.5 ± 5.6), IL-23 (19.3 ± 4.8), IL-6 + IL23 + TGF-β (22.7 ± 2.7) stimulated cells as compared to MLSA (12.3 ± 3.3) alone stimulated cells in T1R patients (Fig. 5E). Of interest, combination of IL6 + TGF-β (14.4 ± 3.1) showed significant down regulation of IL-17A secretion as compared to all combinations in T1R patients. Subsequently, NR patients showed significantly (p < 0.001) high Th17 cells in IL-6 + TGF-β + IL23 (16.3 ± 3.9) group as compared to MLSA (10.7 ± 2.4) stimulated cells alone, but not with other combinations.

**Figure 6:**
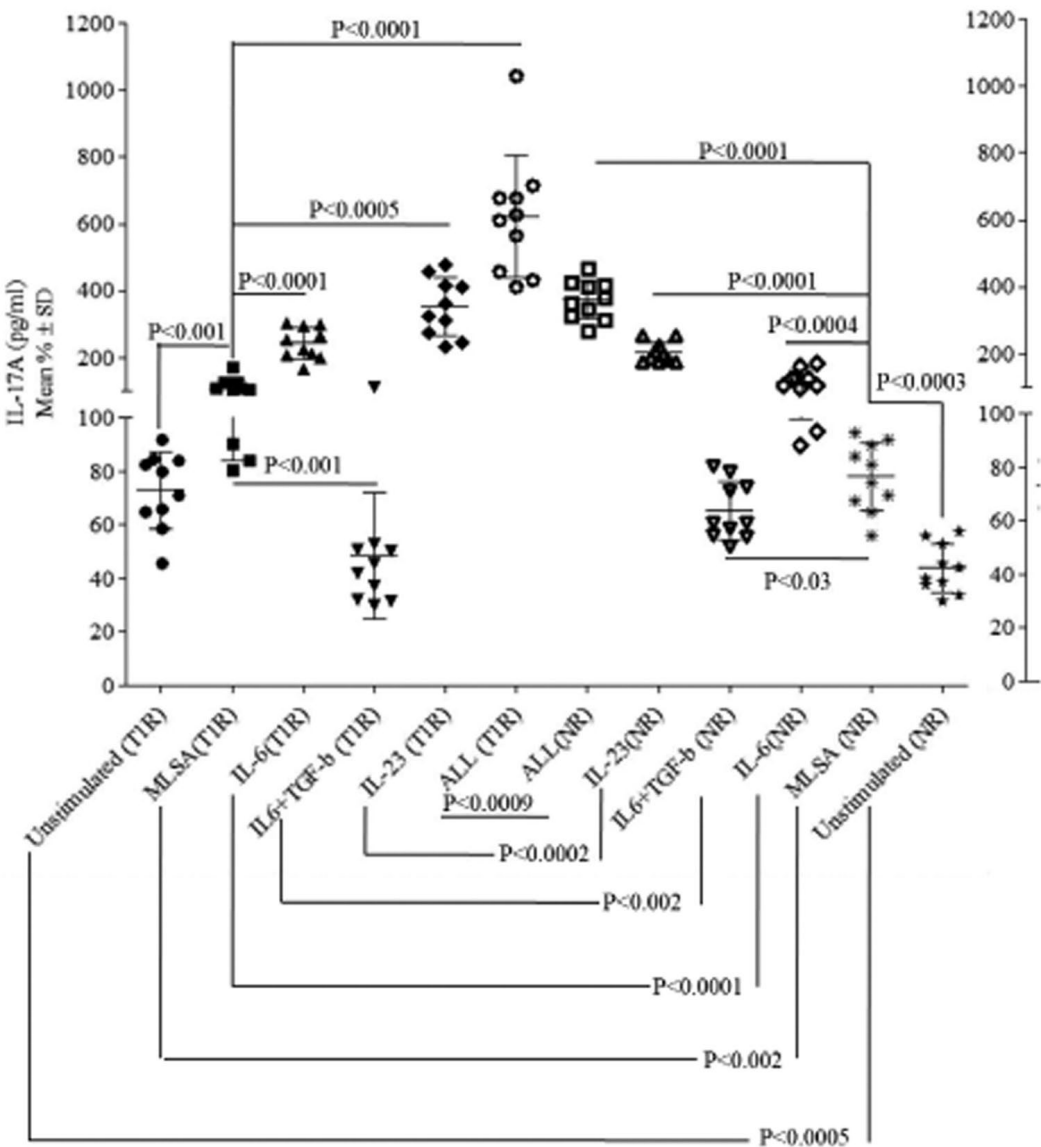
Combination of IL-6 + TGF-β showed significantly down regulated IL-17A protein as compare to all combinations in T1R and NR patients. Representative scatter plot is showing mean% ± SD of IL-17 (pg/ml) in 48 h MLSA and diffrent combinations of recombinants IL-6, IL-23 and TGF-β stimulated culture supernatant in T1R (10) and NR (10). Abbreviations: Mann Whitney T-test was performed for the statistical difference, p < 0.05 value was considered as significant NR; Non-reactions, T1R; Type 1 reactions), MLSA; *Mycobacterium leprae* sonicated antigen.

Moving ahead we next estimated the protein levels of IL-17A by sandwich ELISA in supernatants of all combinations of T1R and NR patients. Figure 6 depicts significantly up-regulated IL-17A (pg/ml) (p < 0.001) in IL-6 (mean% ± SD; 244 ± 47.4), IL-23(352 ± 87.3) and IL-6 + IL-23 + TGF-β (622 ± 183.9) combination supernatants as compared to MLSA alone (110 ± 25.8) within the T1R group. Similarly, NR patients also showed up-regulated IL-17A (p < 0.03-p < 0.0001) in all combinations as compared MLSA alone. Interestingly, the combination of IL-6 + TGF-β showed significantly down regulated IL-17A protein as compare to all other combinations in both NR and T1R patients. We further analyzed these results between the T1R and NR patients. T1R showed significantly high IL-17A (p < 0.002-p < 0.0005) as compared to NR patients. Only (IL-6 + TGF-β) group showed significantly high IL-17A (p < 0.002) in NR as compared to T1R patients (Fig. 6). These results (IL-17 A protein data) thereby fully support our previous flowcytometry data.

### Blocking IL-6R with blocking IL-6R antibody reduces IL-17A production

**Figure 7:**
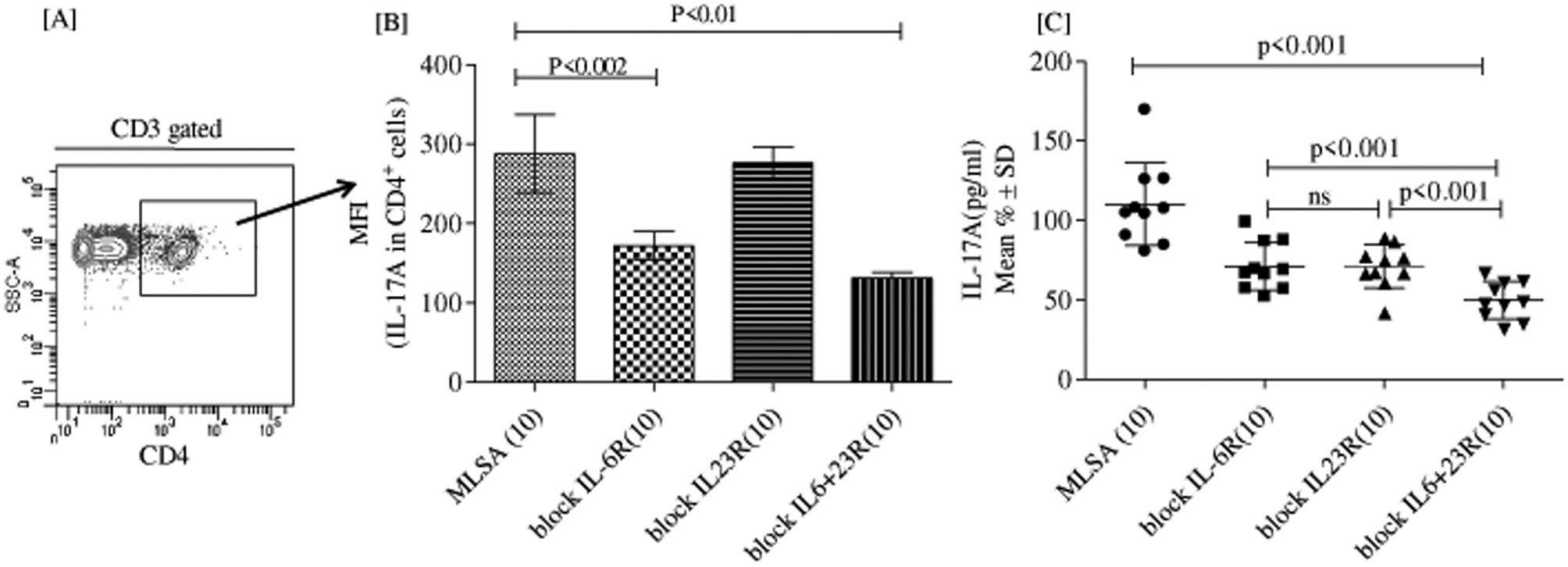
Blocking of IL-6R and IL-23R reduce IL-17A production. (**A**) Representative flow dot plot is showing MLSA stimulated CD3^+^ gated CD4^+^ cells in 48 h cultured PBMCs with and without blocking IL-6R, IL-23R and combination of IL-6R + IL-23R by blocking antibodies. (**B**) Histograms are showing IL-17A MFI (Mean ± SD) in CD4^+^ cells (**C**) Scatergram is showing mean ± SD of IL-17 (pg/ml) in 48 h culture supernatant of MLSA stimulated PBMCs with and without blocking IL-6R, IL-23R and combination of IL-6R + IL-23R by blocking antibodies. Abbreviations: Mann Whitney T-test was performed for the statistical difference, p < 0.05 value was considered as significant. (); Parenthesis shows patients number, NR; Non-reactions, T1R; Type 1 reactions), MLSA; *Mycobacterium leprae* sonicated antigen.

To validate whether enhanced IL-6R expression on CD4^+^ T cells is causative of elevated IL-17A response in T1R leprosy patients, we studied the effect of blocking of IL-6R and IL-23R in MLSA induced IL-17A production by PBMCs cells in-vitro*.* Figure 7 depicts significantly reduced mean florescence intensity (MFI) (p < 0.002) of IL-17A (MFI; 171.7 ± 18) in blocking IL-6R as compared to MLSA (MFI; 287.8 ± 49.4) in CD4^+^ T cells (Fig. 7A,B). Moreover, IL-17A (pg/ml) protein level was also significantly (p < 0.001) decreased in blocking IL-6R cells (71.4 ± 15.3) as compared to (110.1 ± 25.8) MLSA stimulated supernatant of cell cultures respectively (Fig. 7C). Interestingly, blocking IL-23R showed no significant difference as compared to MLSA in cell cultures, but supernatant showed significantly down regulated IL-17A (70.9 ± 13.5 pg/ml) (p < 0.001) as compared to MLSA stimulated supernatant. Interestingly, we also took a group with combination of both blocking-IL-6R and blocking-IL-23R which showed significantly inhibited (MFI; 131.2 ± 17.1) (p < 0.01) MLSA induced IL-17A production by CD4^+^ T cells. IL-17A protein level was further validated by MFI data which is significantly low (49.8 ± 11.7) (p < 0.001) as compared to other combinations. Altogether, these data confirmed that enhancement of IL-6R expression on CD4^+^ T cells accounted, at least partially, for the elevated Th17 responses in patients with T1R.

### Blocking IL-6R and IL-23R with blocking- antibodies induced TGF-β but not FOXP3 expression

**Figure 8:**
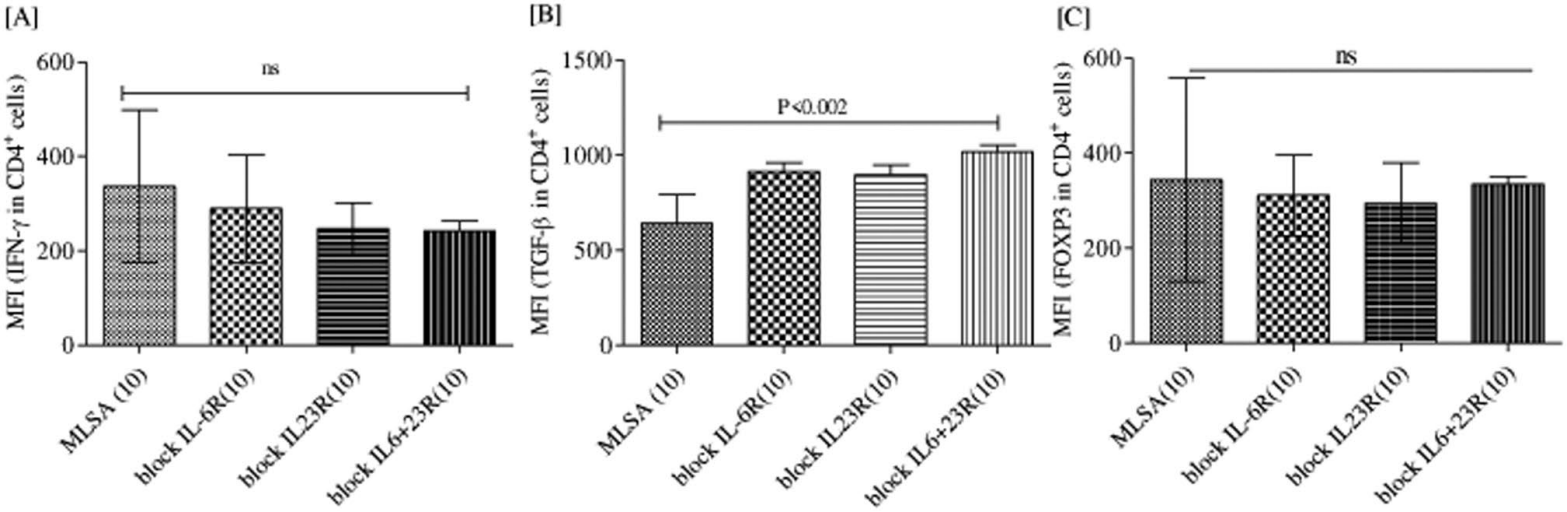
Blocking of IL-6R and IL-23R enhance TGF-β not IFN-γ and FOXP3 expression. Representative histograms are showing CD3^+^ gated CD4^+^ cells in 48 h cultured MLSA stimulated PBMCs with and without blocking IL-6R, IL-23R and combination of IL-6R + IL-23R by blocking antibodies. (**A**) Histograms are showing IFN-γ MFI (Mean ± SD) in CD4^+^ cells (**B**) Histograms are showing TGF-β MFI (Mean ± SD) in CD4^+^ cells. (**C**) Histograms are showing FOXP3 MFI (Mean ± SD) in CD4^+^ cells. Abbreviations: Mann Whitney T-test was performed for the statistical difference, p < 0.05 value was considered as significant. (); Parenthesis shows patients number, NR; Non-reactions, T1R; Type 1 reactions), MLSA; *Mycobacterium leprae* sonicated antigen.

Figure 8 shows MFI of IFN-γ (Fig. 8A) TGF-β (Fig. 8B), FOXP3 (Fig. 8C) in CD4^+^ T cells. Intriguingly, FOXP3 a regulatory cell marker showed no difference after blocking of IL-6R and IL-23R. On the other hand, TGF-β showed significantly (p < 0.002) increased induction after blocking with blocking-IL-6R and blocking-IL23R antibodies. Moreover, no effect of IL-6R blocking with blocking IL-6R on IFN-γ production by CD4^+^ T cells was observed.

## Discussion

The role of inflammatory Th17 cells in pathogenesis have already been explored in many bacterial^27–29^ and viral infections^30^ along with autoimmunity^27^ and cancer^31,32^. Differentiation of inflammatory Th17 cells depends upon a specific cytokine milieu and specific signaling via receptors. Our earlier study Saini et al.^8^ showed that when T helper cells polarization is not occurring against *M. leprae* antigen, Th17 cells play a protective role in stable leprosy (NR) patients. Moreover IL-23 and its receptor are involved in differentiation of Th17 cells in NR patients^8^. *M. leprae* specific T-cell mediated immunity is the main cause of T1R and it has already been assessed by in-vitro studies against *M. leprae* antigens^33^. T1R leprosy reactions occur for clearing bacteria and such increased cellular immune responses may be beneficial because they promote bacterial killing mechanisms. However, the accompanying inflammation in and around the infected nerve tissue can result in severe and irreversible damage and is a matter of debate. Our group earlier reported Saini et al.^18^ that Th17 response was significantly high in T1R patients as compared to NR. Moreover, the present study showed that its not IL-23 and TGF-β but IL-6 and its receptor IL-6R which are high in T1R. Taken together, all these data provided evidence that Th17 cells are involved in cell mediated inflammation. In the present study, we have analyzed 21 genes related to Th17 signatures and involved in inflammation. IL-6R was showed to be significantly high in T1R and correlated with IL-17 isomers. On the other hand, IL-23R was correlated with IL-17 isomers in NR patients but not in T1R patients. These finding also indicates that both IL-6 and IL-6R are important for T1R leprosy reactions. Moreover, two patients have shown high intensity of gene expression as they had erythematous plaques with hyperesthesia and tenderness clinically. The correlation also supports that inflammation occurs due to IL-6 and its receptor in T1R patients.

The present study attempts to investigate the pathway underlying the induced Th17 response in T1R patients, we first observed cytokine milieu which was involved in IL-17 production and found that IL-6 and IL-23 play a major role in immunopathology of T1R patients. Additionally, decreasing or absence of Th1 associated IL-12/IFN-γ promotes IL-23/IL-17 for protective immune response observed in primary infection by *M. tuberculosis* and *M. leprae*^34,35^. IL-6 is a pleiotropic cytokine which can control immune regulation in infectious diseases. In the context of autoimmune disease, IL-6 in combination with TGFβ1 promotes the differentiation of Th17 cells. On the other hand IL-6 also suppresses the differentiation of Treg cells. A report by Heink et al.^36^ showed many sources of IL-6 production via different mechanisms involved in generation of pathogenic Th17 cells and the inhibition of Treg cells in-vivo. Another significant report considered Th17 lineage with protective nature in infectious diseases. Some important reports found that IL-17^+^ cells are a part of the innate T-cell immune response like neutrophils activation during the primary response to intracellular pathogen^9,37,38^. However, findings diverge on the association of traditional CD4^+^IL-17^+^ T cells and showed adaptive immune response to these infections^39^. Many studies showed that IL-17 helps to create bridge between innate and adaptive immune responses with the help IFN-γ. TGF-β plays distinct role during Th17 immune responses in *M. leprae* infection, by assisting the differentiation and production of Th17 responses in leprosy. However, TGF-β is associated with FOXP3^+^ Treg differentiation and stability and also our previous study have already reported that TGF-β is low in T1R as compared to NR leprosy patients^18^. The absolute function of TGF-β in regulating and balancing Th17 and Treg response in T1R patients remains to be elucidated. Subsequently, TGF-β was maintained in homeostatic differentiation of Th17 cells with IL-6. This can be another homeostatic kinetics in T1R and NR patients and it should be explored further. Consistent with these findings, 48 h in-vitro stimulation (MLSA, IL-6 and IL-23) experiments showed that PBMCs produced significant amount of IL-17A. Thus, further investigations are necessary to understand the mechanism underlying the increased IL-6R expression on CD4^+^ T cells, as well as the induced Th17 responses in T1R patients. Subsequently, consistent with our previous findings in patients with T1R leprosy, we found IL-6R and IL-6 expression in stimulated PBMC cells, correlated with the Th17 response in T1R. However, we did not find any difference unlike *M. leprae* antigens which down-regulated IL-6R expression on CD4^+^ T cells from patients with NR patients. Among the IL-6R and IL-23R, increased CD4^+^IL-6R^+^ T cells showed T cell activation via T cell receptor (TCR) engagement or upon binding of IL-6 to IL-6R^40,41^. Because proliferation of *M. leprae* antigen-specific Th17 cells depend on TCR engagement with high IL-6R expression and is correlated with specific IL-17A response in our study, this mechanism explains the increased IL-6R expression in T1R as compared to NR leprosy individuals in this study. Therefore, increased IL-6R expression might be due to engagement of IL-6, the production of which is able to reduce *Mycobacterium* infection^42,43^. More significantly, our in-vitro data showed that blocking of IL-6R signaling on CD4^+^ T cells significantly inhibited non-specific (PHA-stimulated) and MLSA specific production of IL-17A, but not IFN-γ as IL-6 is not involved in Th1 cell differentiation. As expected, TGF-β production was significantly enhanced after blocking with IL-6R and IL-23R along with significantly down regulated IL-17. These findings further validate our previous findings thereby establishing synergistic and opposite regulation between Treg and Th17 cells^18^. We found no significant co-expression between the IL-6R and IL-23R in MLSA stimulated cells (data not shown).Also, these cells are different in nature and further investigation is needed.

In summary, our present study for the first time reports that CD4^+^IL6R^+^ T cells are involved in IL-17A production in T1R leprosy patients*.* Thus, it can be a key mechanism for Th17 responses in patients with T1R. Firstly, CD4^+^IL-6R^+^ and not CD4^+^IL-23R^+^ T cells increase the production of IL-17A in T1R. Secondly, *M. leprae (MLSA)* antigen activates IL-6R to produce IL-17A by CD4^+^ T cells from T1R leprosy patients. Thirdly, the production of IL-17A depends on IL-6R signaling. Thus, understanding the immuno-pathogenic nature of Th17 cell responses in T1R patients is of prime importance, and would thus lead to opening of novel avenues in the treatment and management of inflammatory response which are lethal for T1R patients.

## Supplementary information

Supplementary Figure.

## Data Availability

All relevant data are within the paper.
