# Supplementary figures and images for: Elevated IL-6R on CD4+ T cells promotes IL-6 driven Th17 cell responses in patients with T1R leprosy reactions
Source: Sci Rep. 2020 Sep 15;10:15143. doi: 10.1038/s41598-020-72148-7 (PMC7493991; doi:10.1038/s41598-020-72148-7)

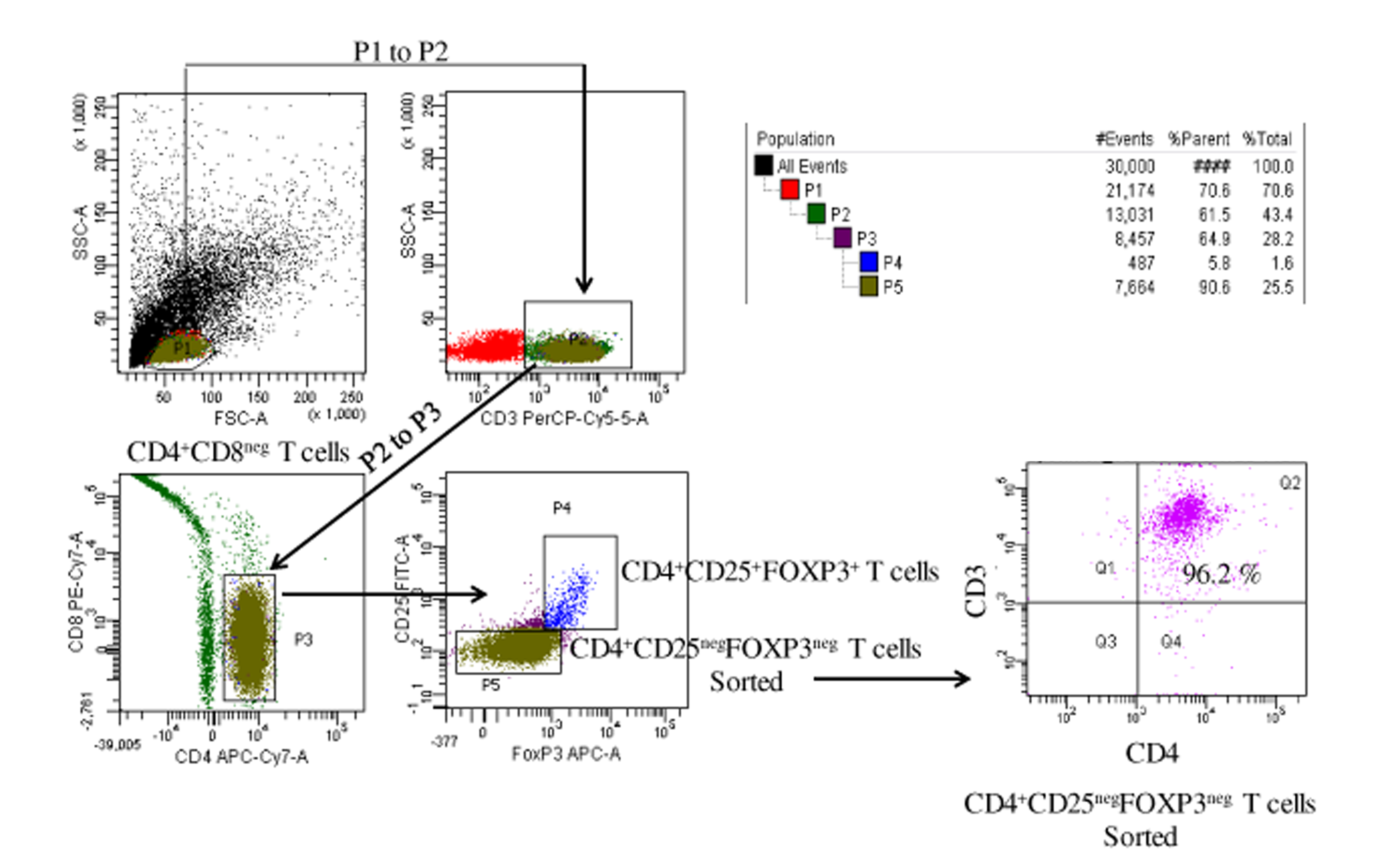

Supplement: Supplementary file 1 — Supplementary Figure. [file 41598_2020_72148_MOESM1_ESM.tif]
